# Awareness, Prevention, Detection, and Therapy Applications for Depression and Anxiety in Serious Games for Children and Adolescents: Systematic Review

**DOI:** 10.2196/30482

**Published:** 2021-12-16

**Authors:** Kim Martinez, Maria Isabel Menéndez-Menéndez, Andres Bustillo

**Affiliations:** 1 Department of History, Geography and Communication University of Burgos Burgos Spain; 2 Department of Computer Engineering University of Burgos Burgos Spain

**Keywords:** serious games, depression, anxiety, children, adolescents, virtual reality, mental health, detection, awareness, prevention, therapy

## Abstract

**Background:**

Depression and anxiety in children and adolescents are major health problems worldwide. In recent years, serious games research has advanced in the development of tools to address these mental health conditions. However, there has not been an extensive analysis of these games, their tendencies, and capacities.

**Objective:**

This review aims to gather the most current serious games, published from 2015 to 2020, with a new approach focusing on their applications: awareness, prevention, detection, and therapy. The purpose is also to analyze the implementation, development, and evaluation of these tools to obtain trends, strengths, and weaknesses for future research lines.

**Methods:**

The identification of the serious games through a literature search was conducted on the databases PubMed, Scopus, Wiley, Taylor and Francis, Springer, PsycINFO, PsycArticles, Web of Science, and Science Direct. The identified records were screened to include only the manuscripts meeting these criteria: a serious game for PC, smartphone, or virtual reality; developed by research teams; targeting only depression or anxiety or both; aiming specifically at children or adolescents.

**Results:**

A total of 34 studies have been found that developed serious games for PC, smartphone, and virtual reality devices and tested them in children and adolescents. Most of the games address both conditions and are applied in prevention and therapy. Nevertheless, there is a trend that anxiety is targeted more in childhood and depression targeted more in adolescence. Regarding design, the game genres arcade minigames, adventure worlds, and social simulations are used, in this order. For implementation, these serious games usually require sessions of 1 hour and are most often played using a PC. Moreover, the common evaluation tools are normalized questionnaires that measure acquisition of skills or reduction of symptoms. Most studies collect and compare these data before and after the participants play.

**Conclusions:**

The results show that more awareness and detection games are needed, as well as games that mix the awareness, prevention, detection, and therapy applications. In addition, games for depression and anxiety should equally target all age ranges. For future research, the development and evaluation of serious games should be standardized, so the implementation of serious games as tools would advance. The games should always offer support while playing, in addition to collecting data on participant behavior during the game to better analyze their learning. Furthermore, there is an open line regarding the use of virtual reality for these games due to the capabilities offered by this technology.

## Introduction

The recent worldwide increase of mental health conditions is a serious health problem. According to the World Health Organization [1], there was a 13% rise in mental health issues from 2007 to 2017. People affected by mental illnesses experience a severe deterioration in their relationships with family, friends, and the community and in daily task performance. The World Health Organization estimates the most common mental health conditions, depression and anxiety, cost the global economy US $1 trillion each year [2]. These issues develop from a very young age; in fact, around 20% of the world’s children and adolescents have a mental health condition. In addition to personal and financial costs, these problems can head to suicide, which is the second leading cause of death among people aged 15 to 29 years [1]. Therefore, research must address this situation by developing resources for mental health education and therapy for the very young.

To address this social demand, research on serious games regarding mental health has emerged in recent decades. Serious games use the ludic medium of video games to achieve learning goals [3]. Their utility resides in creating immersive and entertaining environments built on learning theories and methodologies based on empirical research to maximize education outcomes [4]. Serious games applied to mental health adapt cognitive behavioral therapy (CBT) to the experience allowing exploratory learning and behavior practice [5]. These tools are especially suitable for children and adolescents, since they are familiar with technology and are attracted to graphics and playability [6]. Furthermore, the development and democratization of technology allows the creation of high-quality games for the most common user devices, PCs and smartphones, and the most novel, such as virtual reality (VR) headsets [7].

Serious games have been proposed since the late 1990s for depression therapy, as the review of Li and Foo [8] outlined. Although these first games targeted varied audiences, the vast majority were applied to children and adolescents [9]. However, these first solutions were very rare, and the first reviews on these topics covered a diverse mix of disorders and neurobiological conditions like anxiety disorder, autism spectrum disorder, and attention deficit hyperactivity disorder [10-14]. More recently, serious games that facilitate exposure therapy for phobias use virtual reality to maximize their impact [15].

The standardization of serious games as reliable solutions for anxiety and depression has taken place in the last 5 years, making possible new reviews focused on serious games developed for depression care [16] and anxiety treatment [17,18]. In addition, Villani et al [19] have studied serious games for emotional regulation, an important approach to prevent depression and anxiety.

Gamified technologies for improving mental health have also been considered [20], although these are not the same as serious games. A gamified experience has gaming elements like rewards, characters, and rules but does not focus on playfulness and fun [21]. Finally, the latest reviews document the different psychiatric applications of serious games [22] and the importance of their application in children and adolescents [7,23]. Table 1 shows the main characteristics of these reviews for anxiety and depression—number of games studied, article publication ranges, disorders considered, and game targets—and their main conclusions.

The analysis provided in Table 1 reveals some limitations of the reviews. The first is the low number of studied games, ranging from 5 to 34 but mostly mixing serious games with commercial games or gamified applications. Furthermore, even being recent studies, there is not a novelty inclusion criteria, so they consider articles published as far back as 1989. Second, regarding subject and target, none of these papers focuses on all the applications for depression and anxiety a game could have, from awareness to therapy. Finally, the conclusions of these reviews point out different game design and evaluation issues, but these may be influenced by article limitations. There is a research gap concerning the latest games developed for all possible applications and their specific targets.

This review aims to study serious games applied to depression and anxiety targeting children and adolescents published from 2015 to 2020 and determine if conclusions obtained by the previous studies apply in the same way to new serious games or if this field is evolving. The paper looked for 4 possible applications in mental health: (1) awareness: learning to recognize mental health conditions and erase the stigma associated with help-seeking behavior [24]; (2) prevention: applying emotional regulation strategies in the day-to-day to improve psychological well-being and relationship satisfaction and to avoid mental health issues [25]; (3) detection: finding through game data any mental health conditions or emotional disorders [26]; and (4) therapy: learning CBT techniques to help improve mental well-being and depression or anxiety symptoms for those already diagnosed [27].

**Table 1:** Comparison of published reviews of serious games for mental health.

Review	Number of studies	Years of publication	Subject and target	Conclusions
Dias et al [16]	28 serious games and gamified apps	2007-2016	Depression care for every possible target	The primary aim of researchers is improving treatment engagement, but there is a lack of effectiveness evaluation.
Barnes and Prescott [17]	5 serious games	2011-2016	Therapy for adolescents with anxiety disorders	The research is limited, but the findings suggest games have the potential to reduce anxiety levels in adolescents.
Villani et al [19]	23 serious and commercial games	2007-2017	Video game effects on emotional regulation and mental health well-being for every possible target	An initial guideline to design serious games for emotional or social abilities and the need for building intervention protocols around commercial games.
Vajawat et al [22]	29 serious and commercial games	2008-2020	ADHD^a^, autistic spectrum disorders, eating disorders, posttraumatic stress, impulse control disorders, depression, schizophrenia, dementia, and healthy aging for every possible target	Serious games have great potential but need to explore more applications and targets; review includes the need for standardization of guidelines, more comparison between studies, and the incorporation of VR^b^ and artificial intelligence.
David et al [23]	34 serious games	1989-2014	Mental health promotion and health behavioral change for children and adolescents	Serious games are not ready for dissemination as a stand-alone treatment/prevention strategy but have the potential to serve as valuable clinical tools.
Zayeni et al [7]	22 serious and commercial games	2012-2019	Therapeutic and preventive video games for children and adolescents	Video games can be an effective tool for psychotherapy but there is a lack of effectiveness evaluation.

^a^ADHD: attention deficit hyperactivity disorder.

^b^VR: virtual reality.

## Methods

**Figure 1:**
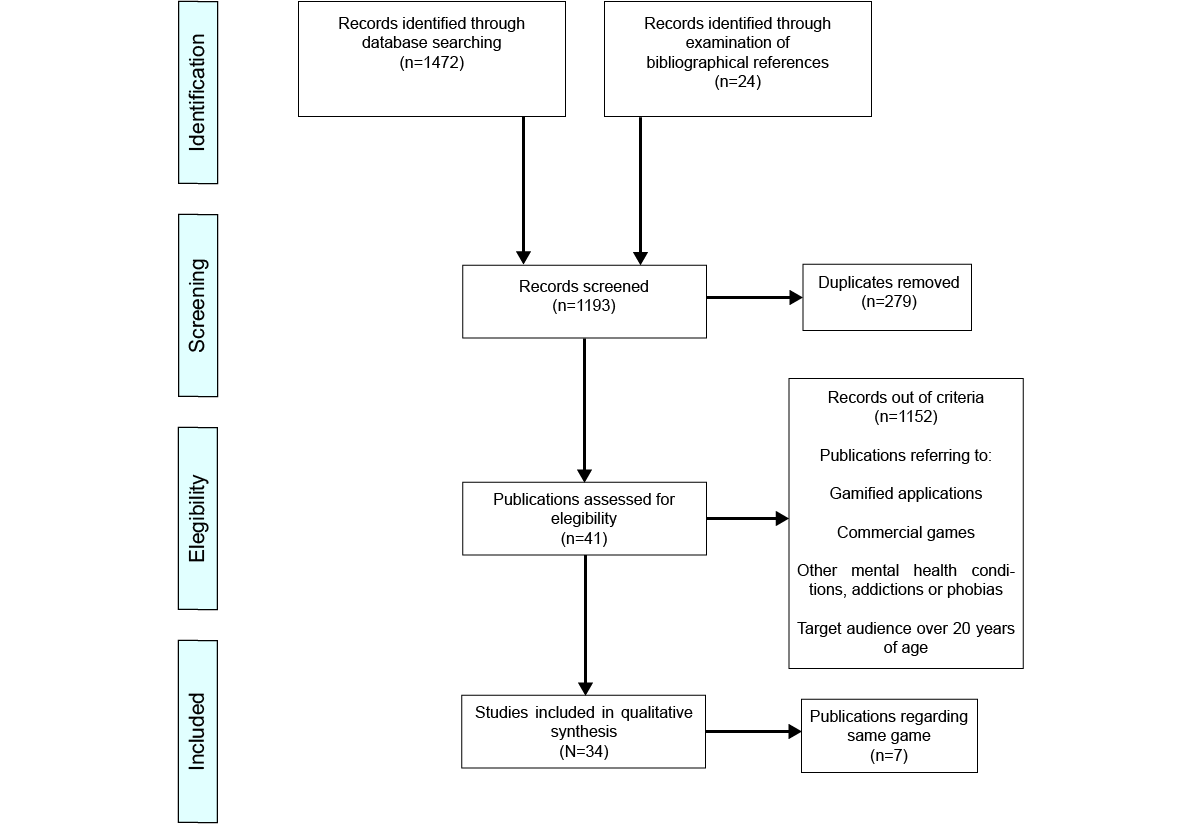
Flow diagram of the selection process.

The serious games selection process was carried out in 4 phases as summarized in Figure 1: identification, screening, eligibility, and inclusion. The identification through literature search was conducted on the following databases: PubMed, Scopus, Wiley, Taylor and Francis, Springer, PsycINFO, PsycArticles, Web of Science, and Science Direct. The years of publication were limited from 2015 to 2020 as previous reviews had studied serious games with similar purposes prior to these dates. The search terms used were “(serious game OR video game OR applied game OR computer game OR mobile game OR online game OR gaming) AND (children OR adolescent OR childhood OR adolescence) AND (cognitive behavioral therapy OR cognitive training OR anxiety treatment OR anxiety disorder OR mental health OR depression OR stigma OR helping behavior OR meditation).” Bibliographical references of the reviews included in Table 1 were also examined and added to this first selection of studies.

The total of 1966 records were identified through this search, and the bibliographical examination added another 24 studies. The following step, as Figure 1 shows, was removing all duplicates, which excluded 381 articles. The remaining 1609 studies were screened to find those studies that considered serious games for depression and anxiety and possible applications to awareness, prevention, detection, and therapy.

Inclusion criteria include games for PC, smartphone, or VR addressing depression or anxiety developed by research teams for children and adolescents. Exclusion criteria include gamified apps; existing commercial games; games for those aged 20 years and older; and games dedicated to other mental health conditions, addictions, or phobias. These criteria were met by only 41 papers.

In cases where the same research team published multiple articles on the same game with the same application, only the most recent papers were selected as these papers were the most developed by the researchers as they had more time for the design and testing and included the highest number of participants. This excluded 7 papers, so 34 studies were included in the qualitative analysis and listed with characteristics in Multimedia Appendix 1 [18,25,28-59].

## Results

For each of the 34 studies, the following information was extracted: game name, mental health condition addressed, application, target age, mental health purpose, year of publication, game genre, tools used for evaluation, type of game performance, devices used, time required, groups of participants, results, and references. From these data, the results were interrelated to obtain the use of serious games and trends in their development.

### Serious Games Applications

**Figure 2:**
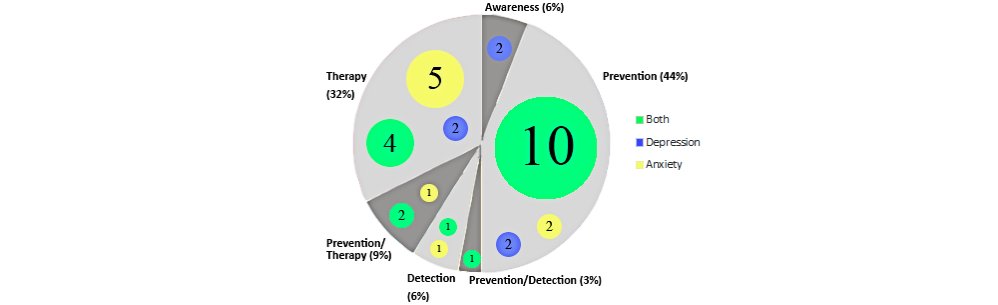
Application of games by mental health condition.

Each game was classified as using 1 of the 4 possible applications contemplated in this review: awareness, prevention, detection, and therapy. The application was also put in relation with the mental health condition to which the game applies, as Figure 2 shows. The majority (30/34, 88%) of serious games serve 1 application. Only 12% (4/34) of games were hybrids mixing 2 applications. The most common (15/34, 44%) application was prevention, followed by therapy (11/34, 32%). On the other hand, awareness and detection were infrequently used, with 2 games each (2/34, 6%). Finally, 9% (3/34) of games used prevention and therapy and 3% (1/34) used prevention and detection.

Figure 2 also shows the relationship between type of application and type of disorder. Of the 34 papers, 26% (9/34) focused on anxiety, 18% (6/34) focused on depression, and 56% (19/34) addressed both issues. Most (10/14, 71%) of the serious games that apply to both mental health conditions were used for prevention. These were games that teach emotional regulation and conflict resolution, skills necessary for a positive mental well-being. It was also common (4/11, 36%) that gaming therapy was equally effective for depression and anxiety since learning CBT was applied for both conditions. Games that exclusively treated depression were equally divided into prevention (2/6, 33%), therapy (2/6, 33%), and awareness (2/6, 33%), which only has cases of this condition. Of the anxiety games, most were used for therapy (5/9, 56%) with the rest for prevention (2/9, 22%), detection (1/9, 11%), and prevention/therapy (1/9, 11%). It stands out that games that treat and prevent anxiety (7/34, 21%) were more numerous than those for depression (4/34, 12%).

### Target Age

The age range for which these serious games are targeted was 6 to 19 years, but there was uneven distribution (Multimedia Appendix 1). Only 3% (1/34) targeted children aged 6 to 7 years, but 21% (7/34) targeted children aged 8 years. Adolescence was the preferred target group of the reviewed papers, especially ages 11 to 14 years (11/34, 32%). The number of games for adolescents aged 18 to 19 years (4/34, 12%) decreased but was still more numerous than for the youngest kids.

Games were targeted to children according to stages of emotional development [60]: (1) young children aged 6 to 7 years—the earliest stage of emotional immaturity; (2) children aged 8 to 10 years—the first phase of development of empathy and self-recognition; (3) children and young adolescents aged 8 to 14 years—the age range that covers the passage from childhood to adolescence and produces more emotional issues; (4) young adolescents aged 11 to 14 years—the second phase of self-recognition based on comparison with the group, decisive in self-esteem; (5) older adolescents aged 15 to 19 years—self-consolidation stage for the achievement of adult maturity; and (6) all adolescents aged 11 to 19 years—the entire adolescence that includes all self-consolidation and emotional regulation learning.

**Figure 3:**
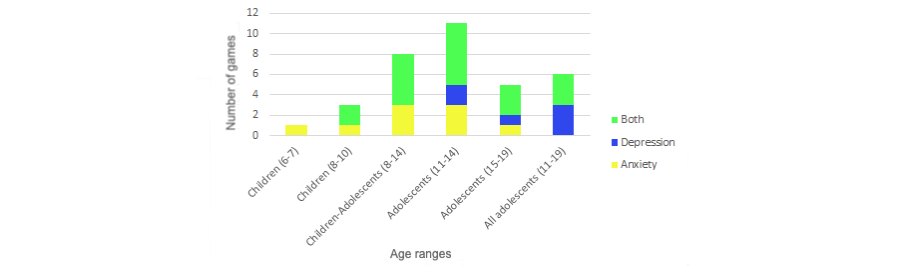
Mental health condition by age range.

Age ranges must be considered when addressing mental health conditions (distribution shown in Figure 3). Anxiety was the only condition treated in children aged 6 to 7 years but was mainly seen in children aged 8 to 14 years. Serious games for depression were only targeted at children aged 11 years and older, and half (3/34, 9%) of these games span the entire range of adolescence. Papers dedicated to both conditions applied to all ages (19/34, 56%) and especially children aged 8 to 14 years (11/34, 32%). The trend that Figure 3 shows is that serious games for anxiety were centered in childhood (5/9, 56%) and games for depression in adolescence (6/6, 100%).

**Figure 4:**
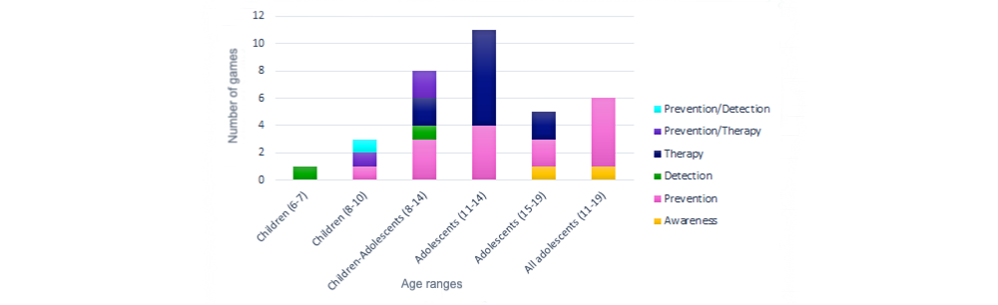
Game application by age range.

The other feature to study regarding age range was game application, as can be seen in Figure 4. Games for detection (2/34, 6%) were limited to childhood, those aged 6 to 7 years, and for children aged 8 to 14 years. In addition, there was a game using detection and prevention for children aged 8 to 10 years (1/34, 3%). On the other hand, awareness games (2/34, 6%) were only targeted to adolescents. The prevention application focused on all age ranges, with its highest percentage in adolescence (11/15, 73%). Therapy also targeted all children, with its highest use on children aged 11 to 14 years (7/11, 64%). Finally, games that mix prevention and therapy were used only in children aged 8 to 14 years (2/34, 6%).

### Mental Health Purpose

**Figure 5:**
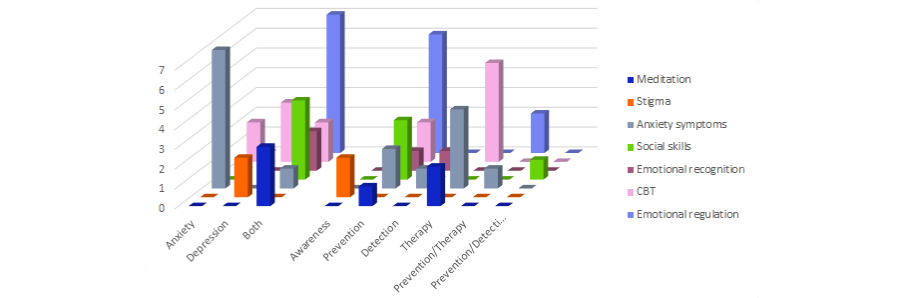
Mental health purpose by its condition and game application. CBT: cognitive behavioral therapy.

Mental health purpose is a characteristic that has been obtained from the qualitative analysis of these serious games. The educational mental health issues that these 34 games address have been classified into 7 categories: training of emotional regulation, emotional recognition, and social skills; reduction of anxiety symptoms and stigma against mental health conditions; CBT; and meditation learning. Concerning mental health conditions, Figure 5 relates the purpose of the game to the issue addressed and highlights how training of emotional and social skills and learning of meditation, necessary skills for positive mental well-being, are dedicated to both anxiety and depression. Logically, games that aimed to reduce anxiety symptoms (8/34, 24%) were targeted at anxiety, except for one that targeted both. However, games aiming to reduce stigma (2/34, 6%) only focused on depression. Finally, games that apply CBT techniques (7/34, 21%) were almost evenly distributed between anxiety and depression.

Comparing mental health purpose with game application, as shown in Figure 5, it is possible to discern the trend that training emotional and social skills was used mainly (10/14, 71%) for prevention. On the other hand, therapy was divided between the learning of CBT and meditation (7/11, 64%) and games aiming to reduce anxiety symptoms (4/11, 36%). These mental health purposes also worked for prevention (5/18, 28%), but to a lesser extent. On the other hand, awareness was completely related to stigma reduction (2/2, 100%). Moreover, the use of detection was divided into emotional recognition (1/3, 33%), social skills (1/3, 33%), and anxiety symptoms (1/3, 33%). In conclusion, the most addressed mental health issues were emotional regulation training (8/34, 24%), anxiety symptoms reduction (8/34, 24%), and CBT application (7/34, 21%).

### Game Genre

**Figure 6:**
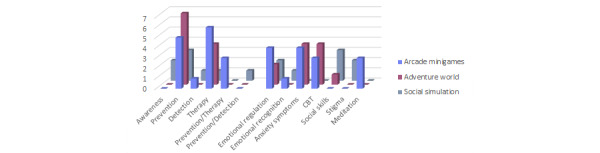
Game genre by game application and mental health purpose. CBT: cognitive behavioral therapy.

Qualitative analysis of the papers allowed them to be organized into 3 game genres: (1) arcade minigames that have short durability and simple playability with various objectives; (2) social simulations of day-to-day environments and issues to learn optimal resolutions and necessary skills; (3) adventure world in which the player takes on the role of an avatar and must interact with missions, characters, and items. This playability characteristic has been put in relation with the game application in Figure 6 to show how these are adapted to serious games. Arcade minigames were used in 44% (15/34) of games, followed by adventure worlds in 32% (11/34) and social simulations in 24% (8/34). Regarding game application, the main ones, prevention and therapy, were equally distributed between arcade minigames (15% [5/34] and 18% [6/34], respectively), social simulations (9% [3/34] and 3% [1/34], respectively), and adventure worlds (21% [7/34] and 12% [4/34], respectively). However, it should be noted that for awareness, only social simulation (2/2, 100%) was used, and adventure world is not a genre applied for detection.

Figure 6 also interrelates the game genre with mental health purpose to continue clarifying the application of gameplay to mental health learning. It is revealed that emotional regulation (8/34, 24%) was the only one that used all game genres. Emotional recognition was addressed with genres arcade minigames (1/2, 50%) and social simulation (1/2, 50%) but not adventure world. Social simulations (8/34, 24%) were used to decrease depression stigma but not anxiety symptoms. Furthermore, CBT learning used the genres arcade minigames (3/7, 43%) and adventure world (4/7, 57%), but meditation only used arcade games (3/34, 9%). Finally, social skills training used the genres social simulations (3/4, 75%) and adventure world (1/4, 25%) .

### Evaluation Tools

**Figure 7:**
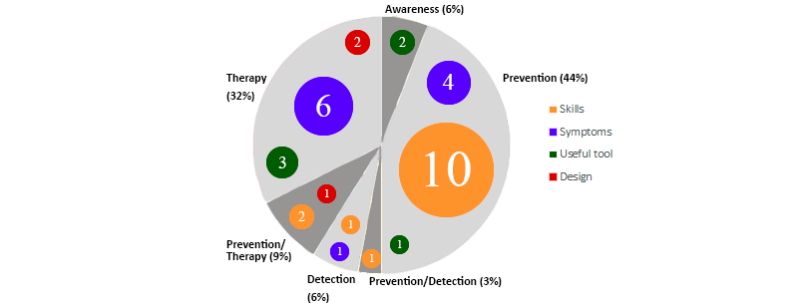
Paper objective by game application.

Assessing the evaluation of the articles result in 3 important issues: objectives of the paper, methods of data collection, and comparison of data. No more variables in common could be found due to the mix of applications and mental health purposes than these serious games address. The qualitative analysis of the papers resulted in 4 possible goals: increase emotional skills, reduce mental health condition symptoms, evaluate game usefulness, and assess game design. The survey analysis shows that 41% (14/34) of games measured skills, 32% (11/34) tested symptoms, 18% (6/34) surveyed the usefulness of the game, and 9% (3/34) evaluated the experience design. Figure 7 interrelates these objectives with the game application to determine the actual focus of this field research. The graphic reveals that most prevention games measured skills (10/15, 67%) and symptoms (4/15, 27%). Detection was also found in these 2 objectives (1/2 [50%] and 1/2 [50%], respectively), while therapy games measured symptoms (6/11, 54%) or verified that it is an effective tool (3/11, 27%). Awareness games aimed to prove that these are useful tools (2/2, 100%) as well. Finally, the studies that were still in the process of assessing game design corresponded to therapy application (3/3, 100%).

**Figure 8:**
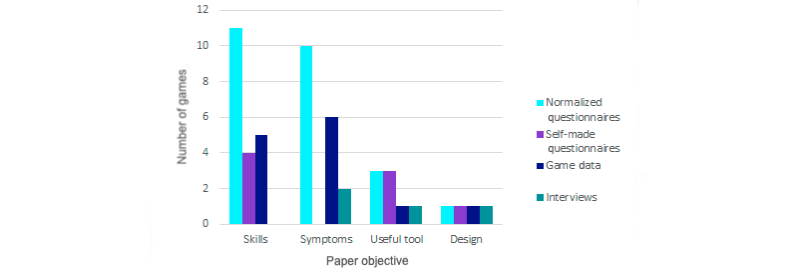
Paper objective by type of evaluation.

Regarding methods of data collection, the qualitative analysis identified 4 tools used in the articles: normalized questionnaires, self-made questionnaires, game data, and interviews. The most common were normalized questionnaires; 74% (25/34) of papers used them to assess changes after the serious game was played. These questionnaires are proven tools in psychology and education that in each case measured symptoms or emotional capacities as appropriate. Furthermore, 24% (8/34) used self-made questionnaires to test their developed experience; 38% (13/34) analyzed game data, and 12% (4/34) conducted interviews with the participants. Figure 8 shows how the data collection tools were distributed according to publication objectives, so the trend to measure the games was revealed. Questionnaires were the most commonly used tools, and this choice stands out to measure skills and symptoms. It is also remarkable within these objectives that normalized questionnaires (25/34, 74%) were used more often than self-made ones (8/34, 24%), except for surveying usefulness (both 3/6, 50%) and checking game design (both 1/3, 33%) when they were chosen equally. The use of game data was highlighted to measure skills (5/14, 36%) and symptoms (6/11, 54%) but was only used once each for useful tool (1/6, 17%) and design assessment (1/3, 33%). Interviews were the least common method, used twice for symptoms (2/11, 18%) and once each for useful tool (1/6, 17%) and design (1/3, 33%).

**Figure 9:**
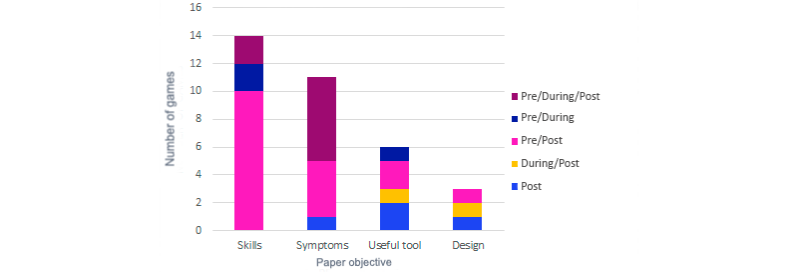
Paper objective by type of data comparison.

Finally, the moment in which the performance data were collected was analyzed: before playing (pre), while playing through the game data (during), and after playing (post). These 3 moments of data collection are combined in the different papers, so there are 5 options: post, during/post, pre/post, pre/during, and pre/during/post. A total of 12% (4/34) of studies performed only postcollection, 6% (2/34) performed during/post comparison, and 9% (3/34) performed the pre/during type. In conclusion, most of the games compared data before and after the serious game. The most common combination (17/34, 50%) was the pre/post evaluation, and 23% (8/34) of studies performed all possible measurements: pre/during/post. Figure 9 shows the relationship of the paper objective with the type of data comparison to get the trend of every measure collection. To measure skills, the most common (10/14, 71%) was a pre-post evaluation, although there were also a few cases of pre/during (2/14, 14%) and pre/during/post comparison (2/14, 14%). The pre/during/post comparison (6/11, 54%) was the most used for symptom measuring, followed by pre/post (4/11, 36%). For the useful tool and design testing, the types of comparisons chosen were similar, but there is no case of pre/during/post evaluation.

### Type of Game Performance

**Figure 10:**
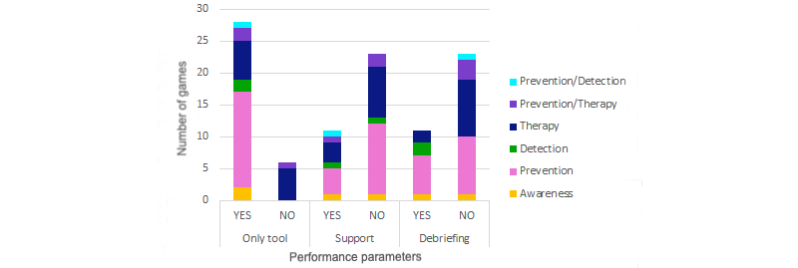
Game performance by game application.

All papers in this review proved the serious games in different ways in their tests so the participants could achieve the set goal. Qualitative analysis discovered 3 parameters that must be taken into account in the performance data: game was used as the only tool to learn so the participants achieve the mental health purpose, participants received support during the game through the gameplay or research assistants, and participants and research assistants performed a debriefing after playing in which participants and educators or psychologists discussed what was played to draw conclusions. The analysis revealed that most of the serious games were performed as the only tool, support was not offered, and debriefing was not performed. Figure 10 shows the distribution of these performance parameters by game application to determine implementation differences depending on the goal of the game. It is important to note that only therapy games (6/8, 75%) used the game as a back tool. Regarding support or lack of it, all applications were proportional in both cases. However, games that did not perform a debriefing were more common in therapy applications (12/14, 86%).

**Figure 11:**
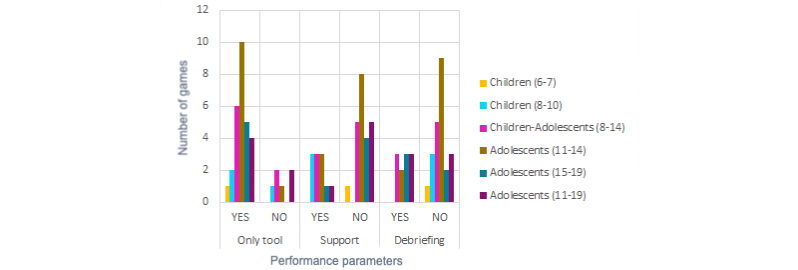
Game performance by age range.

The performance of serious games within the different age ranges is shown in Figure 11 to determine if the games were planned differently depending on the target. The use of the game as an only tool was proportional to the number of games in each range. Support during the game was offered to children (6/12, 50%) more often than to adolescents (5/22, 23%). Debriefing was not offered after any game in children aged 10 years and younger. Debriefing was offered after some games for adolescents aged 11 to 14 years (5/19, 26%) and was much more common (6/11, 54%) for older adolescents.

### Game Devices

**Figure 12:**
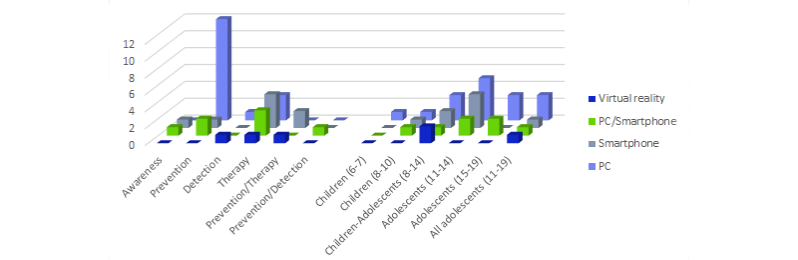
Device used by game application and age range.

Games were developed for PC (16/34, 47%), smartphone (8/34, 23%), PC and smartphone (7/34, 21%), and VR (3/34, 9%). This technical aspect has been related to game application in Figure 12 to reveal potential development trends based on the objective. The most notable trends are that the majority (12/16, 75%) of PC games were dedicated to prevention and most (4/8, 50%) smartphone games to therapy. On the other hand, awareness (2/2, 100%) was mainly used on smartphones (1/2, 50%) and detection (1/2, 50%) on PC and VR.

Figure 12 shows the choice of device according to the targeted age range to discern any possible difference by target maturity. The result is that serious games for children aged 10 years and younger were developed for PC and smartphone equally. For the age range 8 to 14 years, development for PC was slightly preferred (4/8, 50%); in addition, most VR games (2/3, 66%) were targeted to this group. Games were developed for PC more often (16/22, 73%) for adolescents; in fact, no games for those aged 15 to 19 years were developed exclusively for smartphones.

### Game Length

The game tests were performed in different ways with respect to time: 26% (9/34) of games used a single session, 50% (17/34) were played in several sessions, and 24% (8/34) of studies did not indicate the playing time. Among the games that used several sessions, the average was 8.1 (range 4-20). The largest number of sessions was a singularity, since the rest of the games were within the range of 4 to 12 sessions. Regarding the session duration, in which all available data has been included, the average was 39.7 minutes. The shortest test was 5.5 minutes and the longest 120 minutes. Data do not always refer to strict playing time as there were tests in which a debriefing was performed and that process was included in this time.

**Figure 13:**
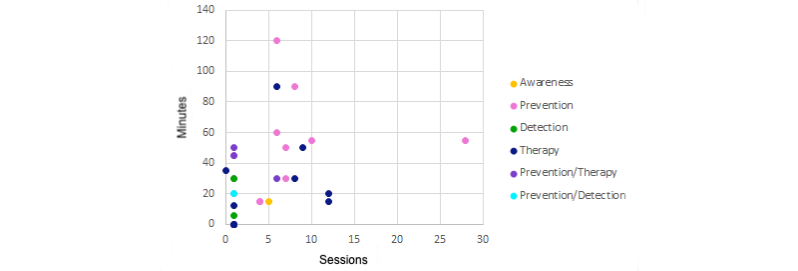
Required time by game application.

The distribution of number of sessions and minutes by game application is shown in Figure 13 to determine possible trends of performance and objective. Any study that did not specify the duration time or number of sessions has been given a value of 0 on its corresponding axis. There is a trend whereby the games with the highest number of sessions and the longest length correspond to prevention, followed by therapy. On the other hand, all detection games included 1 game session of less than 40 minutes. Finally, of the 2 awareness games, one does not give time data and the other did 5 sessions of 15 minutes.

**Figure 14:**
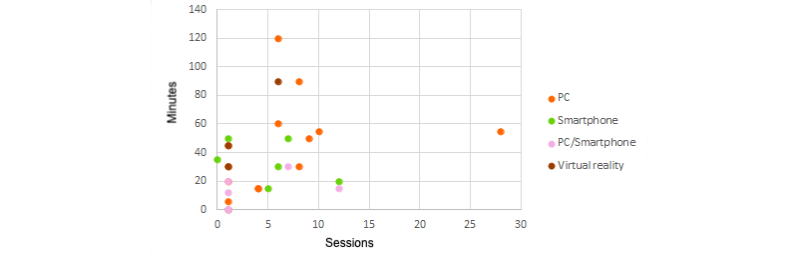
Required time by device used.

The time spent with the device used has been interrelated in Figure 14 to show the possible relationships of time played with technical development. Games that required the highest number of sessions and most length were most often PC games. All games for smartphone or smartphone and PC took less than an hour, although the number of sessions was highly variable. Most VR games (2/3, 66%) used only 1 session of less than 50 minutes.

### Groups of Participants

**Figure 15:**
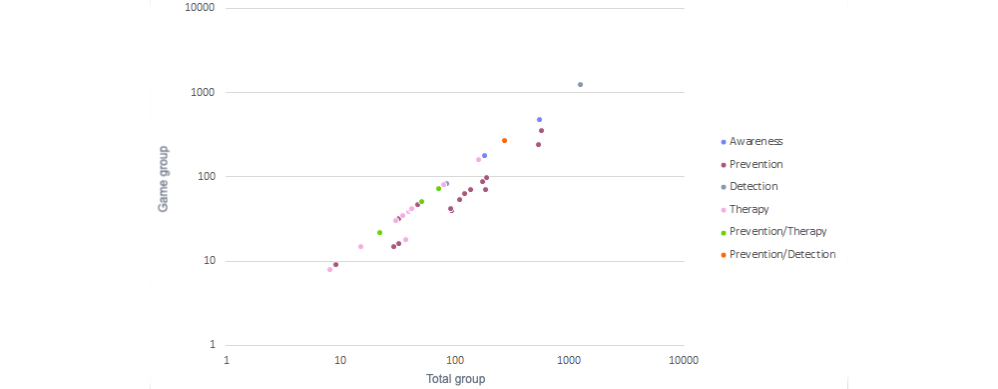
Total groups and game groups by game application.

All games in this review were tested with groups of participants ranging from 8 to 1236 people. The last case was an exception because the game with next highest number had 574 children. The vast majority (29/34, 85%) had fewer than 200 participants, and the average was 156.79. Of the games, 38% (13/34) included a control group while 62% (21/34) evaluated only the group that played the game. The relationship between the total group of participants, the game group, and game application can be seen in Figure 15. The objective was to find a trend between the aim of the games and the number of participants in their tests, in addition to the division between game and control groups.

There was a big difference between therapy groups, which averaged 45.54 participants, and prevention groups, which averaged 157.53 participants. Detection and awareness applications were also tested with large groups. Furthermore, the representation of so many points on the diagonal between both axes shows that most games (20/34, 59%) were tested with the total number of participants. On the contrary, there was a trend in which prevention games (12/15, 80%) usually used control groups.

## Discussion

### Present Trends and Future Lines

#### Implementation of Serious Games

Most games identified treat both anxiety and depression. In addition, there were more games with prevention and therapy applications. This trend is logical since depression and anxiety are closely related and they can lead to each other. Likewise, many therapeutic and emotional management techniques are equally valid for both conditions [61]. Taking a closer look at the results, more games address prevention than therapy. It is true that the main focus of education and psychology should be to avoid suffering mental health issues. Furthermore, these tools can be applied to many participants in school or high school classes. On the other hand, the application of therapy games is smaller since these are logically reserved for children and adolescents already diagnosed.

Continuing with the applications, results reveal that more awareness and detection games are needed as there are only 2 each. It is also noted that, so far, there are only awareness games about depression, probably because these recent games have followed an emerging line of research that fosters emotional awareness to prevent adolescence depression [62]. Ideally, both applications should be mixed since the lack of emotional awareness is equally associated with symptoms of depression and anxiety at these ages [60]. Another option would be to add detection to the development of prevention games. When emotional regulation techniques are being taught, game data can be collected to detect any possible behavior problem or lack of emotional recognition. Likewise, adding awareness to prevention games teaches maintaining a positive well-being while recognizing its importance and erasing mental health stigma.

Another relevant interrelation found in the games implementation is age and mental health condition. Most of these games are dedicated to adolescence but should be applied earlier as children begin to develop depression and anxiety from the age of 3 years [63]. There is also a trend that games for depression are exclusively addressed to adolescence and games for anxiety exclusively to childhood. Once again, this bias may follow the current line of research that focuses depression on adolescents [62], while research on anxiety in children is becoming more relevant [64]. The prevalence of anxiety is as relevant in adolescents as it is in children [61], so this implementation should be fair. Regarding the application, it stands out that awareness games are aimed at adolescents and detection games at children. Given that lack of emotional awareness can foster anxiety and depression symptoms at an early age [64], there should be no differences. Likewise, detection games should be extended to adolescence to avoid the serious development of these mental health conditions [62].

Finally, there is a bias concerning mental health purposes and applications of these serious games. Games that teach emotional regulation, emotional recognition, and social skills are those of prevention, since these skills serve to avoid both conditions [60]. On the other hand, therapy games focus on reducing anxiety symptoms and practicing CBT and meditation. Even so, all these purposes can be used for prevention or therapy. Therefore, a future line should be that more games serve prevention and therapy together, since there are only 3 of the 34 that do this. The only exception is stigma reduction, which is applied only in awareness games. It should also be noted that no game tested depressive symptoms on their own, so this would be an open research field.

#### Development of Serious Games

The game genres developed can be classified into arcade minigames, adventure worlds, and social simulations. Arcade minigames are used most often because the results are short games, easier to adjust to the implementation conditions. Since most tests are finished in an hour, these games can be repeated several times until the objective is achieved, also presenting a challenge so the user does not get bored. The next most popular genre is adventure world, which take advantage of the important ability of serious games to entertain so the learning is acquired. When a plot is played with a main character the user identifies with, this allows the recognition and assimilation of its emotions. Social simulations are used to generate empathy with characters and familiar environments that the users might experience in their real life. In this way, users can be directly taught how mental health conditions affect the lives of people who are diagnosed with them [20].

Regarding the interrelation with applications, awareness games use only social simulations to show the reality of mental health conditions to the participants. Adventure world is not a genre that is used for detection, probably because these are more playful games that focus on the character and not the player, so it is not possible to detect anything. Exploring mental health issues, emotional regulation is the most applicable to the 3 game genres. The techniques applicable in this learning are diverse to adapt them to many types of gameplay. Emotional recognition games do not develop adventure worlds as these do not seem to offer enough first-person interactivity. In addition, social simulation is not a genre in which anxiety reduction or CBT is applied. It is logical because this game genre is based on telling stories, although it is the most ideal for social skills and stigma reduction. Last, meditation is only taught with short arcade minigames as these techniques are performed the same in nondigital scenes.

Concerning devices tendencies, therapy games often take advantage of smartphone facilities. Most of these games have been designed to be played on a day-to-day basis, so it is more comfortable to use a portable device. On the other hand, prevention games are mostly performed in school classrooms where it is easier to access PCs. The way in which the experience is going to be introduced should be assessed when choosing the serious game device to save resources. In any case, it does not seem that the age at which the game is targeted affects this choice. Finally, future research should focus on VR because this review has only found 3 serious games in the recent years. This technology has proven to achieve positive results for learning and measuring user behavior [65].

Another important factor in game development is playing time. The vast majority of the analyzed games are played within 1 hour. This length responds to the duration of a class or a therapy session in which it is usually applied. Games with therapy and prevention applications tend to be longer because participants need more time to learn these techniques. It is also logical that longer games are developed for PC because the device is more comfortable than for smartphone or VR games. For VR, the duration is limited by the lack of familiarity with this device, which can cause dizziness or visual fatigue [65]. Therefore, the decision with this variable must be made according to the developmental capacities of the research team and its application.

It should be noted that certain data were searched for without success: video game development engine, software, applied game design, and availability. The lack of this more technical data was probably due to author specialization. Most of the authors are researchers in education or psychology who had not developed the game themselves. On the other hand, almost every study indicated a multidisciplinary team created the game but did not follow a specific game design. Finally, the search included the free public access to the developed game. Only 3 of the 34 games were available; the vast majority of articles did not indicate this aspect or pointed to websites that were not working. Since one of the great limitations of these studies is the lack of participants [18], it would be important to disclose and give access to serious games so psychologists, educators, or parents could try them. In this way, more data would be obtained, the game design would be assessed, and the ability of serious games to raise awareness, prevent, detect, or help within therapy would increase.

#### Evaluation of Serious Games

In this review, the game performance for the participants has been analyzed. It is highlighted that therapy games are not used as stand-alone tools because these people are already diagnosed and need complete treatment. Regarding the support or debriefing implementation, there is no statistical difference with respect to other applications. However, since the previous reviews find a lack of current and conclusive results of the usefulness of serious games [7,16], it may be better that all applications are not used as stand-alone tools. Serious games should serve as an appealing approach within an educational program for prevention, detection, and awareness of mental health conditions. Ravyse et al [66] determined in their review of different game designs that it was better to offer support through gameplay and perform a debriefing after the game. Therefore, all serious games should aim to offer this complete experience for further learning. Age should not matter, as has been seen in the analysis of the results, although it should be taken into account that children may need more help than adolescents.

Looking at the participant groups, prevention, detection, and awareness games are tested with the largest groups and therapy games with the smallest. It is a logical consequence of participant availability for these evaluations because the first 3 applications are usually performed in schools in which many students can participate. Therapy games, on the other hand, are played with small groups that have had depression or anxiety diagnosed.

Following with tools used for evaluation, most studies measure skills to test if participants improve or symptoms decrease after playing. These 2 evaluations are the most applied for prevention and detection since these verify the learning of techniques or if the game helps to improve their emotional state. Negative postcollection results would indicate if the user has an emotional issue that needs to be addressed. However, therapy games only measure symptoms because these games are used strictly in populations after diagnosis. In addition, awareness and therapy applications are the only games that verify serious games are a useful tool; awareness learning evaluates an immediate change of judgment, and therapy involves a process evaluated by a psychologist.

To measure these capacities, questionnaires are the most widely used tools, especially normalized ones. It is logical that proven tools are chosen, which also provides the opportunity to compare the results of a serious game with other nondigital programs. Even so, this review has not found that the same questionnaire models are used even if the same symptoms or skills are measured. As pointed out in previous studies [22], this should be standardized in the future, allowing for comparison of results between different game genres, age targets, group numbers, playing lengths, and even applications. So far, the evaluation of serious games is so different that a comparison of effectiveness is not possible. Furthermore, game data are evaluated in less than half of the studies, a circumstance that should change. Serious games should be designed to collect data on player behavior and choices. This would be the most objective measure, as questionnaires are affected by the subjectivity of the participant. Last, interviews are a good method of obtaining data, although many times these cannot be performed because of the time they take. It will depend on the researcher’s capacity, but these cases are usually limited to therapy.

Regarding data comparison, 73% of articles evaluated before and after playing. This should be the standard to measure any changes caused by the game playing. Ideally, researchers should collect all types of data: pre-post with questionnaires or interviews and during the experience through game data. This would alleviate one of the problems found in these studies: missing data that prevent clearer results from being obtained [23]. At present, most games that measure symptoms already prefer this pre/during/post comparison. It makes sense that these are the ones that should most closely measure whether the player has a mental health condition. Finally, a summary of these trends and future lines is shown in Table 2.

**Table 2:** Summary of trends and future lines of serious games.

Analyzed processes in this serious games review		
**Implementation**		
	**Trends**	
		The majority of games treat both depression and anxiety, with prevention and therapy being the most common applications.
		Exclusive games for depression are targeted to adolescence and anxiety games to childhood.
		Prevention and therapy games have the widest range of learning: emotional and social skills, meditation, cognitive behavioral therapy, and anxiety symptom reduction.
	**Future lines**	
		More awareness and detection games are needed, and the 4 applications should be mixed to increase the effect.
		Anxiety and depression should be equally addressed in all age ranges.
		There are still no games that test depressive symptoms.
**Development**		
	**Trends**	
		The most common game genres are arcade minigames, adventure worlds, and social simulations, in this order.
		Arcade minigames and adventure worlds can serve most types of learning, while social simulations are more valuable to teach social skills and stigma reduction.
		Therapy games usually use smartphones and prevention games use PC, regardless of the target age.
		Most games are played within 1 hour, but prevention and therapy games can be longer.
	**Future lines**	
		There should be more games for VR to take advantage of the learning capabilities this device offers.
		Game design should be standardized to better develop these games.
		These serious games should be available to the general public to assess design and development.
**Evaluation**		
	**Trends**	
		Therapy games are the only ones not used as a stand-alone tool.
		Prevention, detection, and awareness games are tested with the largest groups, and therapy games with the smallest.
		Most studies measure skills improvement or symptom reduction in participants, commonly using normalized questionnaires.
		Most studies collect and compare participant data before and after playing, which should be the standard.
	**Future lines**	
		Serious games should not be the only tool for the learning and should always offer support and perform a debriefing.
		Studies should use standardized questionnaires so the results could be compared between studies.
		Games should be developed so playing data could be collected and analyzed to understand participant behavior and learning.

#### Comparison With Previous Reviews

One of the conclusions find in previous studies is that the research is limited [17]. The authors of this paper agree with this statement. This review has focused on finding all serious games published between 2015 and 2020, with a broad focus on their application for depression and anxiety in children and adolescents. The number of studies found was 34, which is not a large number. Previous reviews have analyzed between 5 and 34 articles but also included commercial games. Although the results show that more games are being developed each year, there is still much pending research. Nevertheless, this is a relatively new research field that largely depends on the technological advances of recent years. Therefore, it is expected that this development will be strengthened from now on. There are more and more free resources and learning tools that can be applied to research, facilitating workloads.

A lack of standardization of development guidelines has been encountered too [22]. This paper reaches the same conclusion by finding that no article followed a game design theory; all claimed to develop it with a multidisciplinary team. This is not to say that these experts have not used this theory but shows it is not standardized to create more games or compare studies. The review by Vajawat et al [22] affirmed the need for VR development and the incorporation of artificial intelligence into games. Our study has only found 3 VR serious games, a technology that should be studied more for its educational capabilities. In addition, the more artificial intelligence is applied to these games, the better the experiences of children and adolescents can be measured to obtain gameplay data.

Another issue in this research field is the lack of effectiveness evaluations [7,16]. This study found that all articles when testing the serious game found positive results regarding the experience purpose. However, it was impossible to compare and assess these outcomes for several reasons. To begin with, these serious games were dedicated to depression, anxiety, or both conditions, measuring completely different skills or symptoms with different evaluation tools. In addition, the participant groups vary too much in number and conditions to be compared. Because of this, the same type of evaluation must be used to determine effectiveness. In addition, many of the articles confirmed the necessity to do more tests with larger groups taking into account more external aspects that may affect the results.

Only 3 of the 34 games included in this review obtained slight differences in their tests with respect to age. These results are strange since there are games that targeted wide age ranges, from 8 to 14 years or from 11 to 19 years. These are stages in which emotional maturation is critical [60]. Therefore, when therapy techniques are applied or social and emotional skills are learned, older children should have more success as they have greater development. The difference between ages should be one of the future research lines.

Last, there is a claim that serious games are not yet ready for dissemination as an independent treatment/prevention strategy [23]. Having already discussed the research issues of limited studies and lack of standardization in development and evaluation, the authors of this study agree. Nevertheless, results are positive and serious games development is experiencing great advances. In addition, most of these games are developed in teams with increasing experience, so their applications are constantly improving. The next few years will be decisive in implementing serious games such as treatment and prevention for depression and anxiety in childhood and adolescence.

### Conclusions

This review studies 34 serious games published between 2015 and 2020 that target children and adolescents to address depression and anxiety. Analyzing their applications has shown a trend that most games are applied on both conditions for different age ranges. Even so, the exclusive anxiety games focus on childhood and the exclusive depression games focus on adolescence. Across all games, most apply prevention, which is usually played on a PC and tested in the largest groups, together with detection and awareness games. The second most used application is therapy, usually on smartphones, not used as a stand-alone tool, and tested in smaller groups. Prevention and therapy games tend to require sessions of 1 hour to complete; longer tests would use a PC. Regarding evaluation, most tests measure the acquisition of skills or the reduction of symptoms. The most used evaluation tools are normalized questionnaires completed before and after playing.

Furthermore, this paper agrees with the conclusions of previous reviews. This research is still limited, although more games are being developed and technological development will increase. A standardized game design theory is needed so all serious game developments follow comparable guidelines. Evaluation tools should be standardized to improve effectiveness evaluation, and studies should use larger groups of participants. Moreover, there is a lack of learning comparison within the age ranges. Once these games are tested, they should be accessible to the public for its benefit and to obtain more data for research. Resolution of these issues in the coming years will determine the implementation of serious games as treatment and prevention tools.

Finally, this review proposes future lines of serious games development after analyzing the missing applications. More awareness and detection games are necessary; in addition, the ideal way to implement these applications would be by mixing them. Another possible mix of these applications would be with prevention, and more games addressing prevention and therapy together are needed. Furthermore, there is a lack of detection games that measure depressive symptoms. All applications should be equally distributed by age, especially since awareness games for children and detection games for adolescents are missing. Regarding devices, it would be interesting to develop more games in VR due to its demonstrated capacity for learning and user behavior measurement. In addition, within game implementation, it is optimal to offer support to the participants and perform a debriefing. Last, when evaluating learning, normalized questionnaires and game data should be used to compare before, during, and after playing.

## Appendix

Multimedia Appendix 1 List of 34 serious games analyzed in this review.

## Abbreviations

CBT: cognitive behavioral therapy
VR: virtual reality
