# Supplementary material for: Awareness, Prevention, Detection, and Therapy Applications for Depression and Anxiety in Serious Games for Children and Adolescents: Systematic Review
Source: JMIR Serious Games. 2021 Dec 16;9(4):e30482. doi: 10.2196/30482 (PMC8726021; doi:10.2196/30482)
Supplement: Multimedia Appendix 1 [file games_v9i4e30482_app1.docx]

**Multimedia Appendix 1: List of 34 serious games analyzed in this review.**

| **NAME** | **REFERENCE** | **MENTAL HEALTH CONDITION** | **APPLICATION** | **PURPOSE** | **DEVICE** | **AGE** | **TIME** | **GROUP** | **RESULTS** |
| --- | --- | --- | --- | --- | --- | --- | --- | --- | --- |
| GameTeen System | [47] Rodriguez, Rey, Vara, Wrzesien, Alcañiz, Baños & Pérez, 2015 | Both | Prevention/ Therapy | Train emotional regulation skills | Virtual  Reality | 9-14 | 45 min | 28M/23F Game group  (51) | Users learnt emotional regulation to deal with frustration |
| SPARX | [34] Cheek, Fleming, Lucassen, Bridgman, Stasiak, Shepherd, & Orpin, 2015 | Depression | Therapy | Try the game to prove is a useful tool to exercise cognitive behavioral therapy | PC & Smartphone | 12-19 | - | 93M/66F  Game group  (159) | Users could apply learnt therapy |
| Village Voices | [35] Cheong, Khaled, Holmgȧrd, & Yannakakis, 2015 | Both | Prevention | Learn conflict resolution in school settings | PC | 10-12 | 4 sessions x 15 min | 20M/12F Game group  (32) | Users improve their conflict resolutions skills |
| REACH app | [43] Patwardhan, Stoll, Hamel, Amresh, Gary & Pina, 2015 | Anxiety | Prevention/ Therapy | Try the game design to prevent and early intervene anxiety symptoms | Smartphone | 9-10 | 6 sessions x 30 min | 10M/12F Game group  (22) | User liked the game design and anxiety symptoms showed improvement |
| Maya | [32] Carrasco, 2016 | Depression | Therapy | Try the game to prove is a useful complementary tool to learn helpful social and mental health behaviors | PC & Smartphone | 12-18 | 12 min | 15 F  Game group  (15) | The majority of users found the game positive for their treatment |
| Dojo | [48] Scholten, Malmberg, Lobel, Engels & Granic, 2016 | Anxiety | Prevention | Reduce anxiety symptoms through emotional regulation training | PC | 11-15 | 6 sessions x 120 min | 48M/90F Game group  (70) Control group  (68) | All users improve their anxiety symptoms |
| Nevermind | [41] Lobel, Gotsis, Reynolds, Annetta, Engels & Granic, 2016 | Both | Prevention | Improve emotional regulation skills by regulating negative states on stressful situations | PC | 18-19 | 45 min | 38M/9F Game group  (47) | Game helped learning to regulate emotions |
| Happy | [40] Filella, Cabello, Pérez-Escola & Ros, 2016 | Both | Prevention | Learn emotional competencies to resolve social conflicts | PC | 8-12 | - | 301M/273F Game group (351) Control group (223) | Users improve their emotional competencies and reduced anxiety levels |
| Stigma-Stop | [30] Cangas, Navarro, Parra, Ojeda, Cangas, Piedra & Gallego, 2017 | Depression | Awareness | Try the game to prove is a useful tool to reduce stigma towards mental health conditions | PC & Smartphone | 14-18 | - | 280M/272F Game group (484) Control group  (68) | Game served to reduce misconceptions towards mental health conditions |
| ScrollQuest | [59] Tuijnman, Granic, Whitkin & Engels, 2017 | Depression | Prevention | Train emotional regulation to control rejection sensitivity | PC | 14-17 | 20 min | 9M  Game group  (9) | Most players were sensitive to rejection |
| The Fling | [28] Boendermaker, Veltkamp & Peeters, 2017 | Both | Prevention | Train emotional regulation to increase behavioral control | PC | 13-17 | 4 sessions x 15 min | 79M/106F Game group  (70)  Control group  (115) | Game improved behavioral control over time |
| Dojo | [50] Schuurmans, Nijhof, Engels, & Granic, 2017 | Anxiety | Therapy | Treat anxiety disorder through CBT relaxation techniques | PC | 13-14 | 8 sessions x 30 min | 31M/6F Game group  (18)  Control group  (19) | Game improved anxiety symptoms |
| SPARX-R | [44] Perry, Werner-Seidler, Calear, Mackinnon, King, Scott, Merry, Fleming, Stasiak, Christensen & Batterham, 2017 | Depression | Prevention | Learn CBT techniques to prevent depression | PC & Smartphone | 16-17 | 7 sessions x 30 min | 249M/291F Game group  (242)  Control group  (298) | Game reduced depression symptoms |
| Mindful gNATs | [55] Tunney, Cooney, Coyle & O’Reilly, 2017 | Both | Prevention | Teach mindfulness techniques to improve mental well-being | PC & Smartphone | 10-12 | - | 52M/41F Game group  (40)  Control group  (53) | Game improved mental states |
| Zoo U | [58] DeRosier & Thomas, 2018 | Both | Prevention/ Detection | Improve social skills to exhibit positive behavior | PC & Smartphone | 8-10 | 20 min | 154M/116F Game group  (270) | Game identified which children should improve social skills |
| SmartCAT | [46] Pramana, Parmanto, Lomas, Lindhiem, Kendall & Silk, 2018 | Anxiety | Therapy | Try the game’s design to prove is as useful as earlier app | Smartphone | 9-14 | X sessions x 35 min | 35  Game group  (35) | Game’s design is equally usefull to reduce anxiety symptoms |
| Developing Emotional Intelligence | [37] D’Amico, 2018 | Both | Prevention | Improve emotions perception abilities | PC & Smartphone | 8-10 | 30 min | 16M/16F Game group  (16)  Control group  (16) | Game improved emotional competencies |
| Moving Stories | [54] Tuijnman, Kleinjan, Hoogendoorn, Granic & Engels, 2018 | Depression | Awareness | Try the game to prove is a useful tool to reduce stigma towards depression and help-seeking behavior | Smartphone | 12-15 | 5 sessions x 15 min | 180  Game group  (X)  Control group  (X) | Game improved depression awareness |
| Pesky gNATs | [56] van der Meulen, McCashin, O’Reilly & Coyle, 2019 | Therapy | Anxiety | Apply CBT to improve anxiety symptoms or low mood | PC | 6-19 | 9 sessions x 50 min | 39  Game group  (39) | Game improved anxiety symptoms |
| Spock | [57] Cejudo, López-Delgado & Losada, 2019 | Both | Prevention | Improve emotional intelligence and psychosocial adjustment | PC | 17-18 | 10 sessions x 55 min | 45M/47F Game group  (42)  Control group  (50) | Game improved emotional competencies |
| Unnamed | [53] Spengler, Hofer & Busch, 2019 | Anxiety | Detection | Detect power stress through its relationship with implicit power motive | PC | 6-7 | 5.5 min | 46M/39F Game group  (84) | High power motive detection turn into children with elevated power stress |
| REThink | [38] David, Cardoș & Matu, 2019 | Both | Prevention | Try the game to prove is a useful tool to teach emotional control | Smartphone | 10-16 | 7 sessions x 50 min | 51M/58F Game group  (54)  Control group  (55) | The game is more effective than the educational program in which is based |
| RegnaTales | [42] Ong, Lim-Ashworth, Ooi, Boon, Ang, Goh, Ong & Fung, 2019 | Both | Prevention/ Therapy | Train anger manegement | Smartphone | 6-12 | 50 min | 49M/23F Game group  (72) | Game improved skills of anger management |
| DEV-TG | [18] Barnes, 2019 | Anxiety | Therapy | Reduce anxiety symptoms | PC | 13-19 | - | 17M/24F Game group  (41) | Game improved anxiety symptoms |
| Muse/ DayDream/ Wild Divine | [51] Schuurmans, Nijhof, Scholte, Popma & Otten, 2019 | Both | Therapy | Meditate to relax and regulate stress | PC & Smartphone | 10-18 | 12 sessions x 15 min | 9M/6F Game group  (15) | Game improved stress symptoms |
| Quest – Te Withianga | [36] Christie, Shepherd, Merry, Hopkins, Knightly & Stasiak, 2019 | Both | Therapy | Try the game’s design to prove is useful and find needed changes | Smartphone | 10-18 | - | 30  Game group  (30) | Game design got changed since the trials |
| Emotiva  Mente | [31] Carissoli & Villani, 2019 | Both | Prevention | Train emotional intelligence | PC | 14-16 | 8 sessions x 90 min | 20M/101F Game group  (64)  Control group  (57) | Game improved emotions evaluation and expression |
| Hall of Heroes | [39] DeRosier & Thomas, 2019 | Both | Prevention | Train social skills | PC | 9-13 | - | 29  Game group  (15)  Control group  (14) | Game improved social abilities to accept affection an express emotions |
| Aislados | [33] Cejudo, Losada & Feltrero, 2020 | Both | Prevention | Improve subjective well-being and mental health through emotional intelligence skills | PC | 12-17 | 10 sessions x 55 min | 87M/100F Game group  (97)  Control group  (90) | Game improved wellbeing, positive affect and mental health |
| Mindlight | [49] Schoneveld, Wols, Lichtwarck-Aschoff, Otten & Granic, 2020 | Anxiety | Prevention | Reduce anxiety symptoms through training of self-efficacy and internalizing and externalizing problems | PC | 8-12 | 6 sessions x 60 min | 55M/80F Game group  (86)  Control group  (49) | Game improved anxiety symptoms |
| Muse | [52] Schuurmans, Nijhof, Scholte, Popma & Otten, 2020 | Both | Therapy | Meditate to relax and regulate stress | Smartphone | 10-18 | 12 sessions x 20 min | 80  Game group  (X)  Control group  (X) | Still waiting for game results |
| Sparky | [45] Pine, Sutcliffe, McCallum & Fleming, 2020 | Both | Therapy | Reduce stress, anxiety and depression symptoms | Smartphone | 13-15 | - | 42  Game group  (42) | Game reduced stress symptoms and increased mental health interest |
| Deusto-e-motion1.0 | [26] Lázaro, Amayra, López-Paz, Martínez, Pérez Alvarez, Berrocoso, Al-Rashaida, García, Luna, Pérez-Núñez, Rodriguez, Fernández, Parada Fernández, Oliva-Macías, 2020 | Both | Detection | Detect social disorders through expression recognition | Virtual Reality | 8-12 | 30 min | 639M/597F Game group  (1236) | Game detected children with low social skills and also improved emotional recognition |
| DEEP | [29] Bossenbroek, Wols, Weerdmeester, Lichtwarck-Aschoff, Granic & van Rooij, 2020 | Anxiety | Therapy | Reduce anxiety symptoms and disruptive classroom behavior | Virtual Reality | 12-17 | 6 sessions x 90 min | 7M/1F Game group  (8) | Game reduced anxiety symptoms and improved behavior for most participants |
